# S‐1 plus cisplatin with concurrent radiotherapy for locally advanced thymic carcinoma: Study protocol of LOGIK1605/JART‐1501

**DOI:** 10.1111/1759-7714.13319

**Published:** 2020-02-05

**Authors:** Minoru Fukuda, Soichiro Funaki, Takuya Yamazaki, Shuntaro Sato, Hiroshi Mukae, Mitsuhiro Takenoyama, Junya Fukuoka, Kazuki Nabeshima, Hisashi Tateyama, Kazuto Ashizawa, Masaki Hara, Takashi Seto, Meinoshin Okumura, Kenji Sugio

**Affiliations:** ^1^ Clinical Oncology Center Nagasaki University Hospital Nagasaki Japan; ^2^ Department of General Thoracic Surgery Osaka University Graduate School of Medicine Osaka Japan; ^3^ Department of Radiology Nagasaki University Graduate School of Biomedical Sciences Nagasaki Japan; ^4^ Clinical Research Center Nagasaki University Hospital Nagasaki Japan; ^5^ Department of Respiratory Medicine Nagasaki University Graduate School of Biomedical Sciences Nagasaki Japan; ^6^ Department of Thoracic Oncology National Hospital Organization Kyusyu Cancer Center Fukuoka Japan; ^7^ Department of Pathology Nagasaki University Graduate School of Biomedical Sciences Nagasaki Japan; ^8^ Department of Pathology, Faculty of Medicine Fukuoka University Fukuoka Japan; ^9^ Department of Pathology Kasugai Municipal Hospital Kasugai Japan; ^10^ Department of Clinical Oncology Nagasaki University Graduate School of Biomedical Sciences Nagasaki Japan; ^11^ Department of Radiology Nagoya City West Medical Center Nagoya Japan; ^12^ Osaka Toneyama Medical Center Osaka Japan; ^13^ Japanese Association for Research on the Thymus (JART) Osaka Japan; ^14^ Department of Thoracic and Breast Surgery Oita University Faculty of Medicine Oita Japan; ^15^ Lung Oncology Group in Kyusyu (LOGiK) Fukuoka Japan

**Keywords:** Cisplatin, radiotherapy, S‐1, thymic carcinoma

## Abstract

Thymic carcinoma is a rare epithelial tumor of the thymus with a poor prognosis, and multimodal approaches are important for its treatment. Recently, a number of studies have indicated that S‐1 treatment is effective against thymic carcinoma. S‐1 plus cisplatin with concurrent radiotherapy is a commonly used treatment for other malignancies, including non‐small cell lung cancer (NSCLC). In addition, its safety has been confirmed, and it has been reported to have a marked effect against thymic carcinoma. Therefore, we conducted a phase II study of S‐1 plus cisplatin with concurrent thoracic radiotherapy for locally advanced thymic carcinoma, in which the overall response rate was employed as the primary endpoint. The secondary endpoints were overall survival, progression‐free survival, and safety.

## Introduction

Thymic carcinoma is a rare epithelial tumor of the thymus with a poor prognosis. Surgery is the mainstay of treatment in resectable cases, and multimodal approaches are playing an increasingly important role in subtotally resected or unresectable cases,^1^ but few studies have evaluated third‐generation drugs or the concurrent use of chemoradiotherapy because of the rarity of these tumors. S‐1 (TS‐1; Taiho Pharmaceutical Co. Ltd., Tokyo, Japan) is a third‐generation oral fluoropyrimidine antitumor agent, which combines tegafur, gimeracil, and oteracil potassium in a molar ratio of 1.0:0.4:1.0, and has been reported to be effective against various solid cancers, including gastric, colon, and non‐small cell lung cancer.^2^, ^3^, ^4^ Recently, a number of studies have indicated that S‐1 treatment is effective against thymic carcinoma.^5^, ^6^, ^7^ S‐1 plus cisplatin with concurrent radiotherapy is a commonly used treatment for non‐small cell lung cancer. Its safety has been confirmed,^8^, ^9^ and it has been reported to have a marked effect against thymic carcinoma.^10^ Therefore, we conducted a phase II study of S‐1 plus cisplatin with concurrent thoracic radiotherapy for locally advanced thymic carcinoma.

## Methods

### Patients

The study protocol was reviewed and approved by the protocol committee of the Lung Oncology Group in Kyusyu (LOGiK) and The Clinical Research Review Board in Nagasaki University (CRB7180001) (registration number: jRCTs071180046). Written informed consent was obtained from all study participants. This study was performed by an independent collaborative (unsponsored) group. It has also been registered with the University Hospital Medical Information Network (UMIN) in Japan (registration number: UMIN000024643).

### Study design and patients

**Figure 1:**
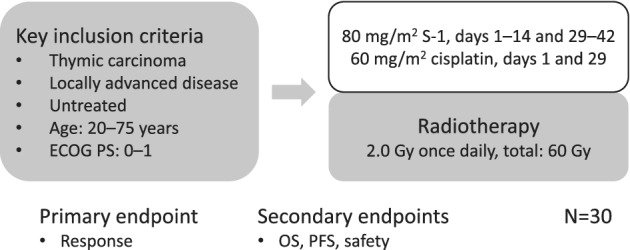
Study schema.

LOGIK1605/JART‐1501 (Fig 1) was initially designed as a phase II, single‐arm trial of S‐1 plus cisplatin with concurrent radiotherapy as a first‐line treatment for patients with locally advanced thymic carcinoma in two groups of LOGiK and Japanese Association for Research on the Thymus (JART).

The eligibility criteria for this study were as follows: A histologically and/or cytologically confirmed diagnosis of thymic carcinoma; not having previously undergone chemotherapy, radiotherapy, or surgery for thymic carcinoma; not being indicated for radical surgical resection; having locally advanced Masaoka stage III or IV disease with or without lymph node metastasis, without distant metastasis and/or dissemination; not experiencing postoperative recurrence; being eligible for radical irradiation according to the treatment plan developed by a radiotherapist; not having any other active malignancies; being aged ≥20 and ≤75 years old; having an Eastern Cooperative Oncology Group (ECOG) performance status of ≤1; having adequate bone marrow function (a leukocyte count of ≥3000/μL or a neutrophil count of ≥1500/μL, a hemoglobin level of ≥9.0 g/dL, and a platelet count of ≥10.0 × 10^4^/μL); having alanine aminotransferase and aspartate transaminase levels of <100 IU/L; having a serum bilirubin level of ≤1.5 mg/dL; having a creatinine clearance rate of ≥60 mL/min; exhibiting arterial O_2_ pressure of ≥60 Torr or SpO_2_ of ≥90%; providing written informed consent; and having a life expectancy of greater than three months. The exclusion criteria were as follows: Having severe complications, such as uncontrolled angina; having suffered a myocardial infarction within the past three months; suffering severe heart failure or an infection; having uncontrolled diabetes or hypertension; having interstitial pneumonia as determined by a chest X‐ray; or having medical problems that were severe enough to prevent compliance with the protocol.

### Assessment

Before treatment, each patient's complete medical history was taken, and all patients underwent a physical examination, assessments of complications, blood cell counts, blood biochemistry tests, a chest X‐ray, chest and abdominal computed tomography (CT), a radionuclide bone scan or positron emission tomography (PET)‐CT, brain magnetic resonance imaging, and electrocardiography. Treatment responses were determined according to the Response Evaluation Criteria in Solid Tumors (RECIST) version 1.1. All adverse events were recorded and graded according to the Common Terminology Criteria for Adverse Events (CTCAE) version 4.0.

### Treatment

The patients received chemotherapy with S‐1 (80 mg/m^2^) in two daily doses after meals on days 1–14 and 29–42, and cisplatin (60 mg/m^2^) as an intravenous infusion on days 1 and 29. The dose of S‐1 was determined according to the body surface area (BSA) of the patient as follows: BSA: <1.25 m^2^, 80 mg per day; BSA: 1.25 m^2^ to <1.50 m^2^, 100 mg per day; and BSA: ≥1.5 m^2^, 120 mg per day. As for the radiotherapy regimen, 2.0 Gy was administered once daily, five times a week, for a total dose of 60 Gy from day 1. Intensity‐modulated radiotherapy was not permitted. The area of the primary tumor on CT was defined as the gross tumor volume (GTV) primary, and metastatic lymph nodes that were enlarged on CT (≥1 cm in shortest diameter) and/or were determined to be positive on PET/CT were defined as the GTV nodal. The volume obtained by adding subclinical extensions of about 0.5 cm to the GTV primary and GTV nodal and then adding the lymph node region containing the GTV nodal was defined as clinical target volume 1 (CTV1). However, no preventative irradiation was administered to the lymph node area downstream of CTV1. The width of the margins was reduced, as appropriate, after considering the patient's anatomical and pathological characteristics. From 40 Gy onwards, CTV2 was obtained by adding subclinical extensions of about 0.5 cm to the GTV primary and GTV nodal. If the protocol treatment markedly reduced the size of the tumor, and it was decided that the tumor was now clinically resectable, the patient was referred for surgical resection.

### Pathological specimens and images

The initial pathological diagnosis was obtained at each facility and confirmed by a central review committee after the treatment had been completed. Similarly, the initial imaging‐based staging and response assessments were performed at each facility and were confirmed by a central review committee.

### Statistical analysis

The primary endpoint of this study was the response proportion, which was assessed by an independent review committee. The secondary endpoints included overall survival, progression‐free survival, and safety. The estimated required number of patients for an accurate binomial test was determined to be >27 based on the following assumptions: threshold response rate P0 = 0.45, expected response rate P1 = 0.70, one‐sided α = 0.05, and ß = 0.20. As some cases might be ineligible, the target sample size was defined as 30. The enrollment period was set at three years, and the follow‐up period was scheduled to last for 1.5 years.

## Discussion

Thymic carcinoma is a rare aggressive neoplasm. Although complete surgical resection is the main treatment for such tumors, it is not always achievable because of local invasion into neighboring organs, diffuse pleural or pericardial invasion. Cisplatin is a key agent for chemotherapy against thymic carcinoma and was included in almost all of the reported regimens for thymic carcinoma, such as adriamycin/cisplatin/vincristine/cyclophosphamide (ADOC) or cisplatin/etoposide, etc. Theoretically, chemotherapy with concurrent radiotherapy seems to be ideal for locally advanced cases, as is the case in other malignancies; however, few prospective studies of treatments for thymic carcinoma have been performed. S‐1 was confirmed to be effective against thymic carcinoma in case reports and retrospective studies,^5^, ^6^, ^7^ in addition, a phase II study for previously‐treated thymic carcinoma has recently been reported.^11^ Overall response and disease control rates were 25% and 75%, respectively. S‐1 plus cisplatin with concurrent radiotherapy has been reported to have a marked effect against thymic carcinoma.^10^ S‐1 plus cisplatin with concurrent radiotherapy is commonly used to treat other malignancies and safety is established; therefore, this phase II study of S‐1 plus cisplatin with concurrent thoracic radiotherapy for locally advanced thymic carcinoma is expected to promote further study of this treatment.

## Disclosure

Dr Fukuda reports personal fees from ONO Pharmaceutical Co., Ltd, grants and personal fees from MSD K.K., personal fees from Chugai Pharmaceutical Co., Ltd., personal fees from Daiichi Sankyo Company, Ltd, personal fees from Kyowa Kirin Co., Ltd., personal fees from Roche Diagnostics K.K, grants from AstraZeneca K.K., grants from Eli Lilly Japan, outside the submitted work; Dr Mukae reports personal fees from MSD K.K., personal fees from Pfizer Japan Inc., grants and personal fees from Boehringer Ingelheim Japan, personal fees from Astellas Pharma Inc., personal fees from AstraZeneca K.K., grants and personal fees from Shionogi &Co., Ltd., grants and personal fees from Daiichi Sankyo Company, Ltd, grants and personal fees from Taisho Pharma Co., Ltd., grants and personal fees from Meiji Seika Pharma Co., Ltd., personal fees from SRL, Inc., grants and personal fees from Asahi Kasei Pharma Corporation, personal fees from Eli Lilly Japan, personal fees from ONO Pharmaceutical Co., Ltd., grants and personal fees from Kyorin Pharmaceutical Co., Ltd., grants and personal fees from Sumitomo Dainippon Pharma Co., Ltd., grants and personal fees from Taiho Pharmaceutical Co., personal fees from Mitsubichi Tanabe Pharma Corporation, grants and personal fees from Chugai Pharmaceutical Co, personal fees from Teijin Home Healthcare Ltd., personal fees from Toa Shinyaku Co., Ltd., personal fees from Nihon Pharmaceutical Co Ltd., personal fees from Janssen Pharmaceutical K.K., grants and personal fees from Fujifilm Toyama Chemical Co., Ltd., outside the submitted work; Dr Takenoyama reports grants and personal fees from AstraZeneca, grants and personal fees from Bristol‐Myers Squibb, grants and personal fees from Chugai Pharmaceutical, grants and personal fees from Covidien Japan, grants and personal fees from Eli Lilly Japan, grants and personal fees from Kyowa Hakko Kirin, grants and personal fees from MSD, grants and personal fees from Nippon Boehringer Ingelheim, grants and personal fees from Novartis Pharma, grants and personal fees from Kaketsuken, personal fees from Pfizer Japan, outside the submitted work; Dr Seto reports grants and personal fees from Astellas Pharma, grants and personal fees from AstraZeneca, grants and personal fees from Chugai Pharmaceutical, grants and personal fees from Eli Lilly Japan, grants and personal fees from Kissei Pharmaceutical, grants and personal fees from MSD, grants and personal fees from Nippon Boehringer Ingelheim, grants and personal fees from Novartis Pharma, grants and personal fees from Pfizer Japan, grants and personal fees from Takeda Pharmaceutical, personal fees from Bristol‐Myers Squibb, personal fees from Kyowa Hakko Kirin, personal fees from Nippon Kayaku, personal fees from Ono Pharmaceutical, personal fees from Roche Singapore, personal fees from Taiho Pharmaceutical, personal fees from Thermo Fisher Scientific, personal fees from YakultHonsha, grants from Bayer Yakuhin, grants from Daiichi Sankyo, grants from Eizai, grants from LOXO Oncology, grants from Merck Serono, outside the submitted work. The other authors have nothing to disclose.
